# Dose-Guided Hybrid AI Model with Deep and Handcrafted Radiomics for Explainable Radiation Dermatitis Prediction in Breast Cancer VMAT

**DOI:** 10.3390/cancers17233767

**Published:** 2025-11-26

**Authors:** Tsair-Fwu Lee, Ling-Chuan Chang-Chien, Lawrence Tsai, Chia-Hui Chen, Po-Shun Tseng, Jun-Ping Shiau, Yang-Wei Hsieh, Shyh-An Yeh, Cheng-Shie Wuu, Yu-Wei Lin, Pei-Ju Chao

**Affiliations:** 1 Medical Physics and Informatics Laboratory of Electronics Engineering, National Kaohsiung University of Science and Technology, Kaohsiung 80778, Taiwan; tflee@nkust.edu.tw (T.-F.L.); cclcnunu@gmail.com (L.-C.C.-C.); i114152106@nkust.edu.tw (L.T.); c108152105@nkust.edu.tw (C.-H.C.); sguy6666@gmail.com (P.-S.T.);; 2 Graduate Institute of Clinical Medicine, Kaohsiung Medical University, Kaohsiung 80708, Taiwan; 3 Department of Medical Imaging and Radiological Sciences, Kaohsiung Medical University, Kaohsiung 80708, Taiwan; 4 Department of Medical Imaging and Radiological Sciences, I-Shou University, Kaohsiung 82445, Taiwan; 5 Department of Radiation Oncology, E-DA Hospital, Kaohsiung 82445, Taiwan; 6 Department of Radiation Oncology, Columbia University, New York, NY 10032, USA; 7 Department of Radiation Oncology, Kaohsiung Veterans General Hospital, Kaohsiung 81341, Taiwan; 8 Department of Radiation Oncology, Kaohsiung Chang Gung Memorial Hospital, Chang Gung University College of Medicine, Kaohsiung 83342, Taiwan

**Keywords:** breast cancer, volumetric modulated arc therapy, radiation dermatitis, deep learning radiomics, ensemble learning, explainable artificial intelligence

## Abstract

Purpose: To improve the prediction accuracy of radiation dermatitis (RD) in breast cancer patients undergoing volumetric modulated arc therapy (VMAT), we developed a hybrid artificial intelligence (AI) model that integrates deep learning radiomics (DLR), handcrafted radiomics (HCR), clinical features, and dose–volume histogram (DVH) parameters, aiming to enhance the early identification of high-risk individuals and support personalized prevention strategies. Methods: A retrospective cohort of 156 breast cancer patients treated with VMAT at Kaohsiung Veterans General Hospital (2018–2023) was analyzed; 148 patients were eligible after exclusions, with RD graded according to the RTOG criteria. Clinical variables and 12 DVH indices were collected, while HCR features were extracted via PyRadiomics. DLR features were derived from a pretrained VGG16 network across four input designs: original CT images (DLR_Original_), a 5 mm subcutaneous region (DLR_Skin5mm_), a planning target volume with a 100% prescription dose (DLR_PTV100%_), and a subcutaneous region receiving ≥ 5 Gy (DLR_V5Gy_). The features were preselected via ANOVA (*p* < 0.05), followed by Boruta–SHAP refinement across 11 feature sets. Predictive models were built via logistic regression, random forest, gradient boosting decision tree, and stacking ensemble (SE) methods. Explainability was assessed via SHapley Additive exPlanations (SHAPs) and gradient-weighted class activation mapping (Grad-CAM). Results: Among the 148 patients, 49 (33%) developed Grade ≥ 2 RD. The DLR models outperformed the HCR models (AUC = 0.72 vs. 0.66). The best performance was achieved with DLR_V5Gy_ + clinical + DVH features, yielding an AUC = 0.76, recall = 0.68, and F1 score = 0.60. SE consistently surpassed single classifiers. SHAP identified convolutional DLR features as the strongest predictors, whereas Grad-CAM focused attention on subcutaneous high-dose regions, which was consistent with the clinical RD distribution. Conclusions: The proposed hybrid AI framework, which integrates DLR, clinical, and DVH features, provides accurate and explainable predictions of Grade ≥ 2 RD after VMAT in breast cancer patients. By combining ensemble learning with XAI methods, the model offers reliable high-risk stratification and potential clinical utility for personalized treatment planning.

## Simple Summary

Radiation dermatitis (RD) is the most common acute side effect of breast cancer radiotherapy and often leads to discomfort, reduced quality of life, and potential treatment interruption. Traditional prediction methods based only on clinical factors or dose–volume parameters are limited in accuracy. In this study, we introduce a novel dose-guided deep learning radiomic approach that specifically analyzes the skin regions that actually receive ≥ 5 Gy of radiation (DLR_V5Gy_). By integrating this innovative feature design with handcrafted radiomic data, clinical data, and dose–volume metrics, we developed an ensemble artificial intelligence model that achieves superior predictive accuracy. Furthermore, explainable AI techniques, including SHAP and Grad-CAM, were applied to validate how the model makes decisions, ensuring transparency and clinical trust. This pioneering framework demonstrates that combining dose-guided imaging features with advanced AI not only improves prediction but also provides actionable insights for personalized prevention and treatment planning.

## 1. Introduction

Breast cancer is the most common malignancy among women globally, with a persistent risk of local recurrence even after surgical resection. Radiotherapy (RT) has been proven to effectively reduce recurrence rates, establishing itself as a cornerstone of adjuvant therapy. With advancements in radiotherapy technology, volumetric modulated arc therapy (VMAT) has become widely adopted in breast cancer treatment because of its superior dose control, which balances tumor coverage with normal tissue sparing [1,2]. However, the proximity of the breast target volume to the skin surface renders the skin highly susceptible to radiation exposure, making radiation dermatitis (RD) the most prevalent acute side effect. Grade ≥ 2 RD, in particular, not only impairs patients’ quality of life but also may lead to treatment interruptions, compromising therapeutic efficacy [3,4,5]. Thus, developing accurate tools for predicting RD risk during treatment planning to identify high-risk patients is of significant clinical value for optimizing treatment strategies and enhancing patient well-being.

Current clinical RD risk assessment relies primarily on clinical factors (e.g., age and BMI) and dose–volume histogram (DVH) parameters for modeling. While these provide some reference value, they fail to adequately capture interindividual tissue heterogeneity and microstructural differences, resulting in limited predictive performance [6]. Recent studies highlight this issue; for example, Lee et al. [7] integrated radiomics and dosiomics to predict RD in breast cancer, achieving an AUC of approximately 0.83, indicating that traditional feature fusion struggles to fully reflect the complexity of the dose distribution and tissue response. Similarly, Xiang et al. [8] applied deep dosiomics for RD prediction, achieving an AUC of 0.82, demonstrating the potential of deep features but limited by non-dose-guided region of interest (ROI) selection and insufficient model interpretability, which hinders clinical adoption. This study aims to address these challenges by leveraging dose-guided deep learning (DLR) radiomics combined with multimodal features to increase prediction accuracy and transparency.

In recent years, advanced artificial intelligence (AI) frameworks have increasingly combined traditional handcrafted features, deep learning radiomics, and clinical variables to increase diagnostic precision and interpretability in oncology. For example, Prakashan et al. [9] proposed a sustainable nanotechnology–AI framework to empower image-guided therapy for precision healthcare. Xie et al. [10] demonstrated that integrating radiomic and dosimetric parameters improved the prediction of acute radiation dermatitis in patients with breast cancer. Similarly, Selcuk et al. [11] developed an automated deep learning-based HER2 scoring system for pathological breast cancer images via pyramid sampling to capture multiscale information.

In addition to oncology, recent advances have demonstrated the efficacy of combining deep and handcrafted radiomic features for improved disease classification across multiple clinical domains. Arafa et al. [12] developed a multistage classification model (MSCADMpox) that achieved high diagnostic accuracy for monkeypox detection. Attallah [13] integrated lightweight CNNs with handcrafted features for lung and colon cancer diagnosis, enhancing cross-domain generalizability. Similarly, Ayyad et al. [14] proposed a multimodal MR-based CAD system for precise prostate cancer assessment, whereas Abhisheka et al. [15] reported that combining deep and handcrafted ultrasound features significantly improved breast cancer diagnostic performance. Collectively, these studies reinforce the growing role of multimodal fusion and explainable AI in advancing diagnostic performance, clinical decision support, and translational applicability.

The advent of radiomics has enabled the extraction of high-dimensional quantitative features from medical images, revealing the microstructural and biological characteristics of tumors and surrounding tissues that are not discernible through conventional imaging [16]. Traditional handcrafted radiomics (HCR) relies on predefined mathematical formulas, offering some interpretability but struggling to capture complex nonlinear patterns [17,18], as reported by Jeong et al. [19], where HCR yielded low performance in treatment response prediction (AUC < 0.70). In contrast, DLR employs convolutional neural networks to automatically learn multilevel image features, demonstrating superior predictive power [8]. However, DLR models often face challenges such as overfitting due to small sample sizes, data heterogeneity, and lack of decision transparency, limiting their clinical translation. Ensemble learning, particularly stacking ensembles, enhances model stability and generalizability by integrating multiple classifiers, as demonstrated in medical imaging applications such as Wu et al. [20] for side-effect prediction. Additionally, explainable artificial intelligence (XAI) techniques, such as SHAP (quantifying feature contributions) and Grad-CAM (visualizing attention regions), improve model transparency and clinical acceptance, as emphasized in recent methodological and healthcare-oriented XAI studies [21,22,23].

To address these challenges, this study proposes a hybrid AI framework comprising the following:

**Dose-Guided ROI Design**: The subcutaneous region receiving ≥ 5 Gy (DLR_V5Gy_) was introduced to enhance the alignment of deep features with clinical RD occurrence.

**Multimodal feature integration**: Combining DLR, HCR, clinical factors, and DVH parameters to leverage complementary strengths.

**Ensemble Learning Architecture**: Employing stacking ensembles to improve model stability and generalizability.

**XAI Analysis**: SHAP is utilized to quantify feature contributions, and Grad-CAM is used to visualize attention regions, ensuring decision validity and clinical acceptability.

The objective of this study was to develop and validate an ensemble model that integrates DLR, clinical factors, and DVH parameters to predict Grade ≥ 2 RD risk in breast cancer patients undergoing VMAT. We hypothesize that dose-guided DLR features combined with multimodal data will significantly enhance predictive performance, with interpretability analyses supporting clinical translation.

## 2. Materials and Methods

### 2.1. Overall Research Framework

**Figure 1:**
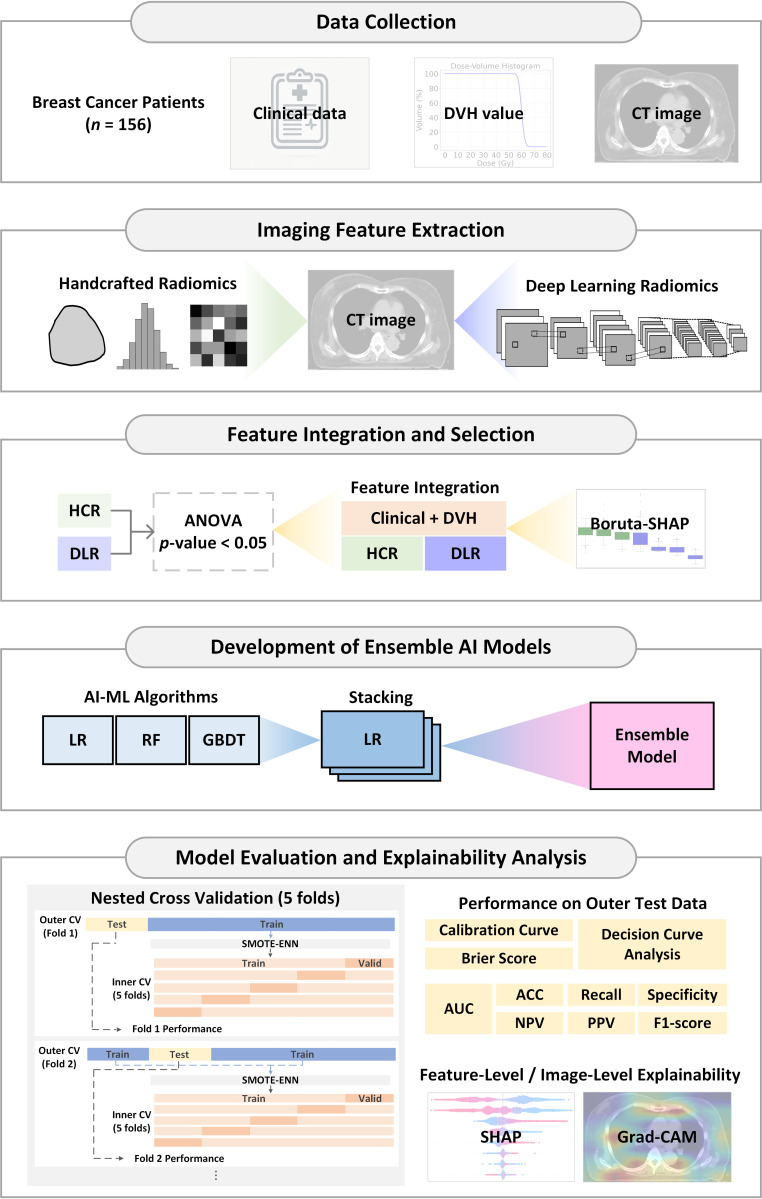
Workflow of the proposed hybrid ensemble artificial intelligence (AI) framework for predicting radiation dermatitis in breast cancer patients. Abbreviation: DVH, Dose-Volume Histogram; CT, Computed Tomography; HCR, Handcrafted Radiomics; DLR, Deep Learning Radiomics; ANOVA, Analysis of Variance; LASSO, Least Absolute Shrinkage and Selection Operator; AI, Artificial Intelligence; ML, Machine Learning; LR, Logistic Regression; RF, Random Forest; GBDT, Gradient Boosting Decision Tree; AUC, Area Under the ROC Curve; ROC, Receiver Operating Characteristic; ACC, Accuracy; NPV, Negative Predictive Value; PPV, Positive Predictive Value; Grad-CAM, Gradient-weighted Class Activation Mapping; Valid, Validation set; SHAP, SHapley Additive exPlanations; SMOTE–ENN, Synthetic Minority Over-sampling Technique combined with Edited Nearest Neighbors.

This study aims to develop a predictive model for RD risk in breast cancer patients undergoing VMAT by integrating HCR, DLR, clinical features, and DVH parameters. The research workflow, as shown in Figure 1, encompasses five main stages:

Data collection
Imaging feature extraction
Feature integration and selection
Development of ensemble AI models
Model evaluation and explainability analysis

### 2.2. Study Population and Data Collection

This retrospective study included 156 breast cancer patients treated with VMAT (prescribed dose: 50 Gy in 25 fractions) at Kaohsiung Veterans General Hospital (Taiwan) between 2018 and 2023. The study protocol was approved by the Institutional Review Board (IRB No. KSVGH23-CT12-09, approval date: 5 December 2023), and all patient data were anonymized in accordance with data protection regulations.

Eligible patients were those with pathologically confirmed breast cancer who received postoperative radiotherapy and had complete clinical, dosimetric, and imaging data, including DVH information and planning CT images enabling extraction of skin and planning target volume (PTV) features. Patients with missing records (n = 6) or insufficient skin region of interest (ROI) volume for reliable radiomic extraction (n = 2) were excluded, yielding a total of 156 analyzable patients.

RD was evaluated by radiation oncologists via the Radiation Therapy Oncology Group (RTOG) criteria, which have been consistently applied in our institution as the standard for toxicity assessment. For binary classification, RD Grade ≥ 2 was defined as a clinically significant (positive) event. All RD grades were documented in the hospital information system to ensure consistency across the 5-year study period.

All patients underwent pretreatment CT simulation (GE Discovery CT590 RT(GE Healthcare, Chicago, IL, USA); slice thickness: 2.5 mm). Treatment plans were generated via Pinnacle3 v9.14 (Philips Radiation Oncology Systems, Fitchburg, WI, USA) or Eclipse v13 systems (Varian Medical Systems, Palo Alto, CA, USA) and delivered via Elekta Synergy or Versa HD linear accelerators (Elekta AB, Stockholm, Sweden). The RT-Structure, RTDOSE, and CT datasets were exported in DICOM format for subsequent feature extraction. Clinical variables (n = 8: age, BMI, laterality, surgery type, AJCC stage, supraclavicular fossa (SCF) irradiation, internal mammary node (IMN) irradiation, and chemotherapy status) and 12 DVH parameters (Skin5mm V5–V50Gy, PTV100%, and PTV105%) were analyzed. The 5 mm subcutaneous region (skin5mm) served as the primary ROI for evaluating the associations between dose distribution and RD occurrence.

### 2.3. Imaging Feature Extraction

To perform radiomic analysis, two types of imaging features were extracted from pretreatment CT images: HCR and DLR.

### 2.4. Handcrafted Radiomics (HCR)

HCR features were extracted via PyRadiomics (version 3.1.0) [24] on the basis of the ROIs delineated in the treatment planning system, adhering to the Image Biomarker Standardization Initiative (IBSI) standards. Each ROI yielded 105 features, including the following:

Shape features: 14

First-order features: 18

Texture features: 73, comprising a gray level co-occurrence matrix (GLCM), gray level run length matrix (GLRLM), gray level size zone matrix (GLSZM), gray level dependence matrix (GLDM), and neighborhood gray tone difference matrix (NGTDM).

### 2.5. Deep Learning Radiomics (DLR)

DLR features were implemented via PyTorch (version 2.6.0, CUDA 11.8, Python 3.9) with a VGG16 convolutional neural network (CNN) as the base architecture. The model utilized ImageNet pretrained weights for transfer learning, leveraging low-level features (edges, textures) learned from large-scale natural images to enhance medical CT image analysis. All 13 convolutional layers of VGG16 were retained with pretrained ImageNet weights and frozen to prevent overfitting. Features were extracted from each convolutional layer, whereas the original fully connected layers were not used. Global average pooling was applied to the convolutional features to generate patient-level deep features.

Model Architecture and Feature Extraction: The VGG16 model retains its original 13 convolutional layers (Conv2d_1 to Conv2d_13). Global average pooling (GAP) was applied after each layer to compress spatial dimensions into channel dimensions, generating feature vectors. The feature counts per layer were 64, 64, 128, 128, 256, 256, 256, 512, 512, 512, 512, 512, 512, 512, totaling 4224 dimensions of deep feature representations. All HCR and DLR features were Z score standardized postextraction.

Image Preprocessing and Input Design: The input images were original-resolution CT slices (512 × 512 pixels) with channel standardization to meet feature extraction requirements. Three-dimensional CT images were processed slice-by-slice, extracting multilevel deep representations. The feature vectors from all the slices of a patient were averaged to generate individual-level DLR feature representations.

**Figure 2:**
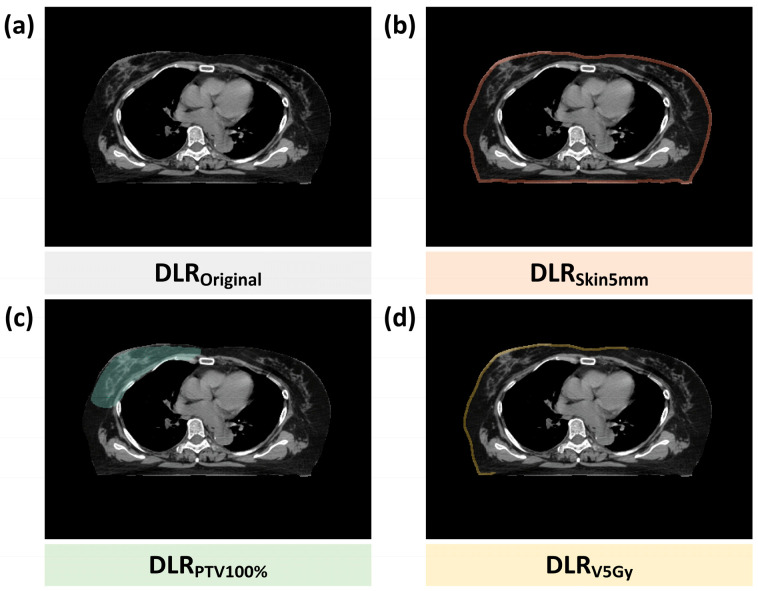
Illustration of DLR input image sources: (**a**) DLR_Original_, (**b**) DLR_Skin5mm_, (**c**) DLR_PTV100%_, (**d**) DLR_V5Gy_. Abbreviation: DLR, Deep Learning Radiomics; ROI, Region of Interest. Colored blocks below each panel represent visual labels for the four DLR input categories and do not convey dose or intensity information.

Four Input Source Designs (Figure 2): To enhance the relevance of features to clinical RD risk, four input strategies were designed:

DLR_Original_: Original CT images without ROI masking, which capture the complete chest wall and adjacent anatomical structures.

DLR_Skin5mm_: 5 mm subcutaneous region, focusing on superficial skin and subcutaneous tissues associated with RD.

DLR_PTV100%_: Planning target volume (PTV) with 100% prescription dose coverage, exploring associations between the primary tumor irradiation region and RD.

DLR_V5Gy_: Subcutaneous region within 5 mm receiving ≥ 5 Gy, excluding nonirradiated tissues to enhance relevance to clinical side effects.

ROI Definition and Dose-Guided Design: The dose-guided ROI (DLR_V5Gy_) was defined as the intersection between the subcutaneous layer within 5 mm of the skin surface and the region receiving ≥ 5 Gy. The 5 Gy threshold corresponds to 10% of the prescribed 50 Gy dose and was selected based on radiobiological and dosimetric evidence indicating that early radiation-induced vascular and inflammatory skin responses arise within this low-to-moderate dose range [25,26]. In accordance with AAPM TG-218 recommendations for patient-specific quality assurance, voxels receiving <10% of the prescription dose were excluded to avoid scatter-dominated noise. Preliminary sensitivity testing with alternative thresholds (3 Gy, 5 Gy, 10 Gy) further confirmed that 5 Gy achieved the optimal balance between irradiated-region coverage and feature stability.

Design Principles and advantages: This DLR strategy balances anatomical information (DLR_Original_, DLR_PTV100%_) and dosimetric effects (DLR_Skin5mm_, DLR_V5Gy_). The DLR_V5Gy_ input, which targets the “actual irradiated skin region,” minimizes noise from nonirradiated areas, improving RD prediction performance. The extracted deep features encompass low-level textures and edges as well as high-level semantic features learned by the multilayer CNN, complementing HCR feature limitations in capturing complex image patterns.

### 2.6. Feature Integration and Selection

To evaluate the contributions of different data modalities to RD prediction, 11 feature sets were designed, covering single sources (clinical, DVH, HCR, or DLR) and multimodal combinations (e.g., clinical + DVH + DLR). This approach not only assesses the predictive potential of radiomics but also clarifies the additive value of clinical and dosimetric data. The detailed feature set definitions and corresponding model performance metrics are provided in Supplementary Tables S1 and S2.

An early feature-level fusion strategy was employed to integrate multimodal data. The clinical, DVH, and radiomic features were concatenated before model training to form a unified feature matrix. All continuous variables were normalized via z score standardization (*StandardScaler*), followed by ANOVA and Boruta–SHAP for feature selection. The fused dataset was balanced via SMOTE-ENN before training the stacking ensemble model. This early fusion approach enables cross-modal learning and ensures balanced feature contributions across modalities.

To mitigate potential bias due to the higher dimensionality of DLR than HCR, the two modalities underwent independent feature selection via an identical two-stage process (ANOVA → Boruta–SHAP). ANOVA (Analysis of Variance): Initial dimensionality reduction was performed via one-way ANOVA (*p* < 0.05) to filter HCR and DLR features significantly associated with RD, reducing redundant variables. Boruta–SHAP: Further refinement was conducted via random forest and SHapley Additive exPlanations (SHAP) values to select features with stable and critical contributions to model decisions. The Boruta–SHAP algorithm was implemented via a random forest–based model without depth limitations (max_depth = None), allowing flexible feature representation. SHAP values were computed via the TreeSHAP algorithm, which was optimized for tree-based learners to estimate feature contributions efficiently. The feature importance estimation was iterated for 200 rounds, with shadow features regenerated at each run to minimize randomness and ensure stable, reproducible identification of important features across folds.

This independent yet standardized design effectively prevented feature importance annihilation between modalities and ensured balanced feature contributions in the subsequent ensemble learning framework. This two-stage process also effectively mitigates overfitting risks and computational burden while enhancing model interpretability and robustness, ensuring that the selected feature sets are statistically significant and clinically relevant.

Additionally, a nested cross-validation (NCV) framework was implemented to ensure rigorous model performance evaluation. Specifically, a fivefold stratified outer loop was used to assess the generalization ability, whereas a fivefold inner loop combined with RandomizedSearchCV was applied for hyperparameter optimization. This nested design minimizes overfitting risk and provides a reliable estimation of generalization in the absence of an external validation cohort.

To mitigate overfitting further, all convolutional layers of the pretrained VGG16 were frozen to limit trainable parameters. Model training and hyperparameter optimization were conducted under the NCV framework to ensure unbiased generalization assessment. Furthermore, a hybrid SMOTE-ENN resampling technique was applied only to the training subsets to balance the class distribution while noisy or ambiguous samples were removed. The final stacking ensemble integrates logistic regression, random forest, and gradient boosting decision tree classifiers to increase model robustness and stability.

Furthermore, the Boruta–SHAP procedure inherently includes an internal stability validation mechanism, ensuring that only robust and consistently important features are retained for model construction. This design enhances both the statistical reliability and clinical interpretability of high-dimensional radiomic analysis.

Dataset partitioning was performed on a per-patient basis to prevent data leakage, ensuring that each patient’s CT, DVH, and clinical data appeared in only one analysis fold. A fivefold stratified outer loop was used to assess generalization, whereas a fivefold inner loop performed hyperparameter optimization via RandomizedSearchCV. Stratification by RD grade maintained balanced class distributions across folds. Clinical characteristics were confirmed to be comparable between the training and testing subsets, with no significant differences observed except for age and internal mammary node (IMN) irradiation.

### 2.7. Development of Ensemble AI Models

To increase the predictive performance and model robustness, a stacking ensemble (SE) approach was adopted, which integrates three complementary base classifiers—logistic regression (LR), random forest (RF), and gradient boosting decision tree (GBDT)—to construct the RD risk prediction model.

The architecture employed a two-layer design:

First layer (base classifiers)**:** LR, RF, and GBDT were trained separately, and the prediction probabilities were output.

Second Layer (Metaclassifier): Logistic regression serves as the metaclassifier, integrating first-layer outputs to generate final predictions. This heterogeneous ensemble strategy enhances the generalization and classification stability.

The model training process included the following steps:

Cross-validation: Nested 5-fold cross-validation was performed on the training set to maintain consistent class proportions across folds.

Class Imbalance Handling: The synthetic minority oversampling technique combined with the edit nearest neighbor (SMOTE–ENN) technique was applied within each cross-validation fold to balance the positive and negative class samples.

Feature Preprocessing: Numerical features are standardized, and categorical features are one-hot encoded.

Hyperparameter Optimization: Randomized search for optimal hyperparameter combinations, followed by retraining on the full training set.

Models were validated on an independent test set to assess real-world predictive performance, implemented in Python via scikit-learn and imbalanced-learn libraries.

To further evaluate component contributions, ablation analysis was performed by independently training and testing each base model (LR, RF, GBDT) and their partial combinations compared with the full SE. This modular structure enabled clear attribution of performance gains to each submodel. Additionally, GBDT hyperparameter sensitivity was assessed to confirm the stability of tree-based learners, which functionally serves as an internal ablation verification.

Logistic regression was adopted as the meta-learner in the stacking ensemble to provide interpretable and stable probabilistic outputs. Unlike nonlinear alternatives such as XGBoost, logistic regression offers transparent weighting of base learner predictions (GBDT, RF, and LR) and minimizes overfitting risk in moderate-sized datasets. Because the base classifiers already capture nonlinear relationships, a linear meta-learner ensures robust aggregation and reliable calibration under nested cross-validation.

### 2.8. Model Evaluation and Explainability Analysis

Multiple performance metrics were used to comprehensively evaluate model classification performance for Grade ≥ 2 RD prediction, with the AUC as the primary metric. Additional metrics included accuracy, recall, specificity, positive predictive value (PPV), negative predictive value (NPV), and F1 score, which were calculated on the basis of the confusion matrix for robust and comparable results. For each performance metric (AUC, accuracy, recall, specificity, PPV, NPV, and F1 score), 95% confidence intervals (CIs) were computed across the outer folds of the nested cross-validation to quantify variability and assess model stability. Between-model comparisons were performed via averaged metrics and 95% confidence interval (CI) analysis derived from the outer folds of the nested cross-validation. Consistent performance gains and nonoverlapping CIs were interpreted as statistically meaningful differences. Additionally, Cohen’s *d* effect size was introduced to quantify the magnitude of practical differences and to evaluate performance stability across folds.

Hyperparameter sensitivity analysis was conducted using results obtained from each inner RandomizedSearchCV iteration within the nested cross-validation framework. The effects of key parameters (n_estimators, max_depth, and learning_rate) were evaluated by summarizing their relationships with the average F1 score to assess model stability and robustness.

To enhance model transparency and interpretability, XAI techniques were employed:

SHAP analysis: SHapley additive exPlanations (SHAPs) quantify the relative contribution of each feature to predictions, establishing feature importance rankings to clarify decision-making [27].

Grad-CAM analysis: Gradient-weighted class activation mapping (Grad-CAM) generates heatmaps to visualize the anatomical or dose-related regions focused on during model inference, validating biological plausibility [28].

By integrating multidimensional performance evaluation and XAI techniques, this study ensures the accuracy, robustness, and clinical validity of the ensemble model, providing a transparent and interpretable AI framework for RD risk prediction.

To assess clinical utility, decision curve analysis (DCA) was performed to estimate the net benefit of the ensemble model across a range of probability thresholds. Additionally, clinical utility metrics, including the PPV, NPV, false positive rate (FPR), and false negative rate (FNR), were calculated at both the default (0.5) and optimal thresholds derived from Youden’s index.

Model performance was assessed via standard classification metrics derived from the confusion matrix: true positives (TPs), false positives (FPs), true negatives (TNs), and false negatives (FNs). The calculation formulas were defined as follows:

(1) $$Accuracy\;=\frac{TP+TN}{TP+TN+FP+FN}$$

(2) $$Sensitivity=Recall=\;\frac{TP}{TP+FN}$$

(3) $$Specificity=\frac{TN}{TN+FP}$$

(4) $$Positive\;Predictive\;Value\;\left(PPV\right)=Precision=\;\frac{TP}{TP+FP}$$

(5) $$Negative\;Predictive\;Value\;\left(NPV\right)=\;\frac{TN}{TN+FN}$$

(6) $$F1\;score=\;\frac{2\times PPV\times Recall}{PPV+Recall}$$

These metrics were calculated for each outer fold of the nested cross-validation to obtain mean values and 95% confidence intervals.

## 3. Results

### 3.1. Study Population Analysis

This study initially enrolled 156 breast cancer patients treated with VMAT. After excluding 6 patients whose clinical data were incomplete and 2 whose ROIs were too small for imaging feature extraction, 148 patients were included in the final analysis. Among these patients, 49 (33.1%) developed Grade ≥ 2 RD during RT, whereas 99 (66.9%) did not reach this severity level.

Table 1 presents the clinical characteristics: the overall mean age was 56 years, with the RD group being significantly older (59 years vs. 55 years, *p* = 0.035). The mean body mass index (BMI) was 24.73, with no significant difference between the groups (*p* = 0.346). A greater proportion of the RD group received internal mammary node (IMN) irradiation (32.7% vs. 17.2%, *p* = 0.033), whereas laterality, surgery type, AJCC stage, supraclavicular fossa irradiation, and chemotherapy use were not significantly different. These findings indicate that age and IMN irradiation are significant clinical risk factors for RD.

### 3.2. Dose–Volume Parameter Analysis

Table 2 summarizes the comparison of the dose–volume features. The RD group presented significantly greater irradiated volumes in the Skin5 mm region across multiple dose thresholds (e.g., V25Gy: 199.8 cc vs. 181.3 cc, *p* = 0.048; V30Gy: 182.5 cc vs. 166.5 cc, *p* = 0.049; V45Gy: 125.4 cc vs. 114.6 cc, *p* = 0.046). Additionally, the RD group had a significantly larger PTV100% (814.5 cc vs. 648.0 cc, *p* = 0.013). These results highlight a dose-dependent relationship between mid-to-high-dose skin exposure and PTV coverage with RD risk, underscoring mid-to-high-dose exposure and target volume as key dosimetric risk factors for RD.

### 3.3. Feature Combination and Selection

To reduce feature dimensionality and eliminate variables not significantly associated with RD risk, an initial screening was performed via ANOVA, *p* < 0.05. For HCR, 160 statistically significant features were selected from an initial pool of approximately 1260 features (based on 105 features per ROI across multiple ROIs, as detailed in Supplementary Table S1).

DLR features were derived from four input sources: original CT images (DLR_Original_), the 5 mm subcutaneous region (DLR_Skin5mm_), the planning target volume with 100% prescription dose (DLR_PTV100%_), and the subcutaneous region receiving ≥ 5 Gy (DLR_V5Gy_), as shown in Figure 2. Each input was processed through 13 convolutional layers of the VGG16 model, generating 4224 feature dimensions. After ANOVA screening, 1153, 544, 723, and 117 significant features were retained for DLR_Original_, DLR_Skin5mm_, DLR_PTV100%_, and DLR_V5Gy_, respectively. These radiomic features were further integrated with 8 clinical parameters and 12 DVH dose metrics to form multiple heterogeneous feature combinations for model construction.

### 3.4. AI-RD Prediction Model Evaluation

Table 3 presents the performance of 11 feature combinations across the four AI models. The details of these feature combinations are provided in Supplementary Table S3. The corresponding Brier scores are reported in Supplementary Table S4. In addition, the results of the statistical significance tests for model comparisons are presented in Supplementary Table S5. For each performance metric (AUC, accuracy, recall, specificity, PPV, NPV, and F1 score), 95% CIs were computed across the outer folds of the nested cross-validation to quantify variability and assess model stability.

Overall, the SE model consistently outperformed the other models:

Baseline combination (Clinical + DVH, Combination 1): AUC = 0.61, indicating moderate predictive power of nonimaging features.

Single Radiomics Modality: DLR_V5Gy_ (Combination 6) achieved the highest performance, with the SE model reaching an AUC = 0.72, demonstrating the high sensitivity of low- to mid-dose skin region features for RD prediction.

Across the NCV outer folds, the stacking ensemble (SE) achieves the highest mean AUC (0.760 ± 0.047) and F1 score, outperforming individual classifiers (logistic regression: 0.705 ± 0.076; random forest: 0.730 ± 0.035; GBDT: 0.500 ± 0.000). Nonoverlapping 95% CIs confirmed statistically meaningful improvements in discrimination and robustness. Cohen’s *d* analysis revealed medium to large effect sizes (|*d*| = 0.7–0.9), with the SE model consistently ranking first across all folds, underscoring its superior and clinically relevant predictive ability.

Multimodal Combination: Combination 11 (Clinical + DVH + DLR_V5Gy_) yielded the best performance, with the SE model achieving an AUC = 0.76, accuracy = 0.70, recall = 0.68, specificity = 0.71, NPV = 0.82, PPV = 0.55, and F1 score = 0.60, indicating superior high-risk identification and low-risk exclusion capabilities.

Sensitivity analyses of key hyperparameters indicated consistent model performance, with only minor changes in average F1 scores across different parameter combinations. These findings confirm that the proposed ensemble framework is robust to hyperparameter variations and maintains stable predictive behavior. The results from the hyperparameter sensitivity analysis of the best-performing combination (Clinical + DVH + DLR_V5Gy_) further confirm the stability and robustness of the proposed model, as shown in Supplementary Figure S1.

The corresponding calibration curves and decision curves for these three representative feature sets are provided in Supplementary Figures S2 and S3.

The DCA and clinical utility assessment demonstrated that SE achieved greater net benefit and superior trade-offs between sensitivity and specificity than single classifiers did. These results confirm that the proposed framework provides practical clinical decision-support potential under varying probability thresholds.

### 3.5. Model Explainability Analysis

#### 3.5.1. Grad-CAM Visualization Analysis

**Figure 3:**
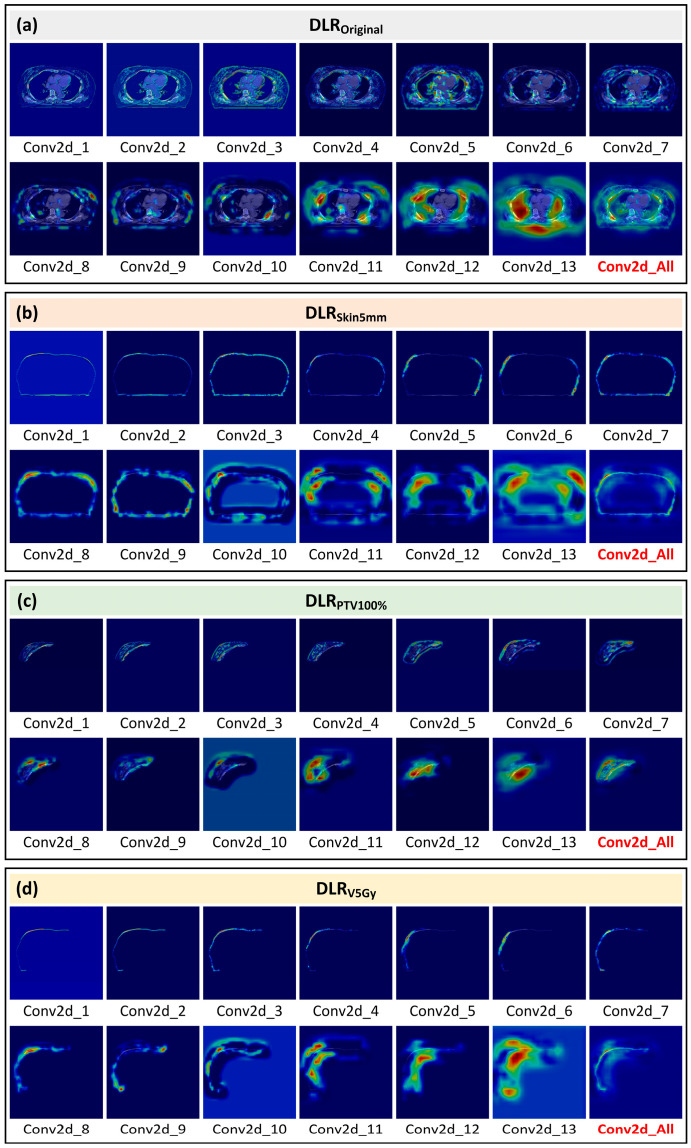
Grad-CAM visualization of DLR features across VGG16 convolutional layers. (**a**) DLR_Original_: unmasked thoracic CT image. (**b**) DLR_Skin5mm_: contoured 5-mm subcutaneous skin layer. (**c**) DLR_PTV100%_: overlap between the 100% prescription-dose region and the 5-mm skin layer. (**d**) DLR_V5Gy_: intersection of the 5-mm skin layer with the region receiving ≥ 5 Gy. Warm colors indicate higher model attention, whereas cool colors indicate lower attention. Block and font colors are used for visual grouping only and do not convey quantitative information. Abbreviations: DLR, Deep Learning Radiomics; VGG16, Visual Geometry Group 16; Grad-CAM, Gradient-weighted Class Activation Mapping.

Figure 3 shows the Grad-CAM response distributions for DLR_Original_, DLR_Skin5mm_, DLR_PTV100%_, and DLR_V5Gy_ across VGG16 convolutional layers. DLR_Original_ showed attention shifting from the shallow-layer chest wall to deeper thoracic structures; DLR_Skin5mm_ focused on dorsal and lateral subcutaneous regions in deeper layers; DLR_PTV100%_ concentrated on tumor boundaries and the core; and DLR_V5Gy_ exhibited early-layer focus on the skin surface, aligning closely with mid- to low-dose regions.

**Figure 4:**
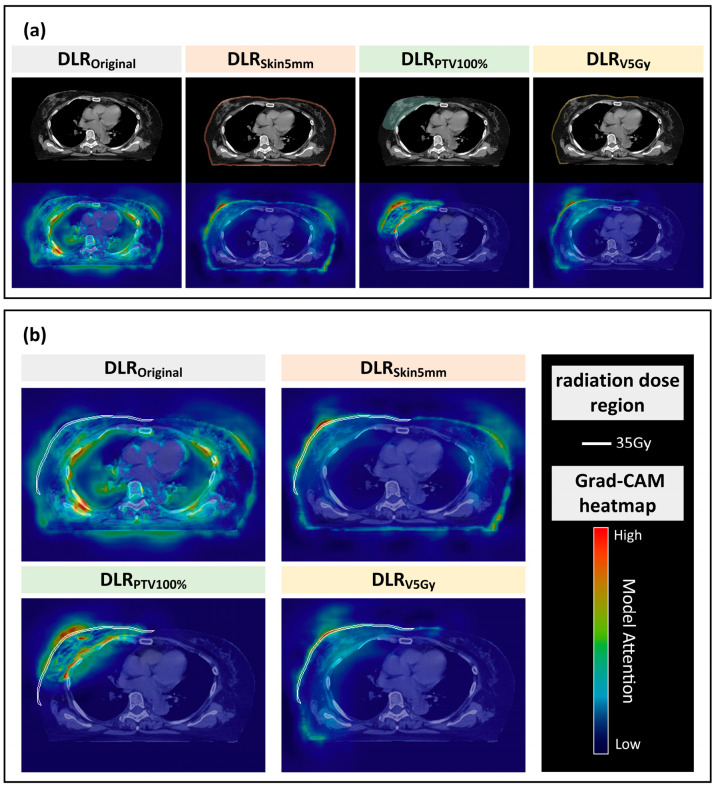
(**a**) Effect of ROI input design on the spatial attention patterns of DLR models across different deep-learning feature inputs. (**b**) Spatial correspondence between Grad-CAM hot-spots and high-risk dose regions associated with radiation dermatitis (RD). The Grad-CAM heatmaps are color-coded from blue to red, where blue indicates low model attention, red indicates high attention, and intermediate green–yellow colors represent moderate attention. Block colors beneath the panels are used only for visual grouping and do not represent dose or image intensity. Abbreviations: DLR, Deep Learning Radiomics; ROI, Region of Interest; PTV, Planning Target Volume; Grad-CAM, Gradient-weighted Class Activation Mapping.

Figure 4 further compares the impact of ROI input design on model spatial attention (Figure 4a) and correlates model heatmaps with high-risk RD dose distributions (Figure 4b). Notably, DLR_V5Gy_ demonstrated the best overlap with RD high-risk dose regions, capturing clinically significant radiation injury patterns and enhancing model credibility.

#### 3.5.2. SHAP Analysis

**Figure 5:**
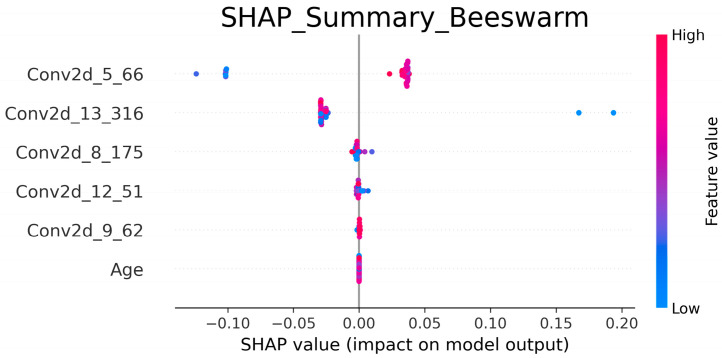
SHAP summary plot for Feature Combination 11 (Clinical + DVH + DLR_V5Gy_). Each point represents a SHAP value for an individual patient, illustrating both the magnitude and direction of the feature’s influence on the model output. Features are ranked by overall importance from top to bottom. The color scale represents normalized feature values, where red indicates high values and blue indicates low values. Positive SHAP values indicate increased predicted risk of radiation dermatitis; negative values indicate reduced risk. Abbreviations: DLR, Deep Learning Radiomics; DVH, Dose–Volume Histogram; SHAP, SHapley Additive exPlanations.

For optimal Combination 11 (Clinical + DVH + DLR_V5Gy_), SHAP analysis revealed that deep learning convolutional features (e.g., Conv2d_5_66, Conv2d_13_316, Conv2d_8_175) were the primary contributors, with stable SHAP value distributions (Figure 5). Clinical variables and DVH features had relatively limited contributions, with only age retained in the model but with low impact. Comparing SHAP results across feature combinations, models relying solely on HCR or DVH features showed increased dependence on clinical variables but significantly lower overall predictive performance. In contrast, incorporating DLR_V5Gy_ features shifted model decisions toward deep image features, underscoring deep radiomics as the critical driver of RD prediction.

## 4. Discussion

This study proposed a hybrid ensemble model that integrates DLR, HCR, clinical variables, and DVH parameters to predict the risk of RD in breast cancer patients undergoing VMAT. Models relying solely on clinical and DVH features achieved limited discrimination (AUC = 0.61), whereas incorporating HCR features provided a moderate improvement (AUC = 0.66). In contrast, DLR-based feature sets achieved consistently higher predictive performance (AUC = 0.70–0.74), indicating that deep learning–derived representations capture more discriminative imaging patterns. Notably, the dose-guided DLR_V5Gy_ feature subset (subcutaneous 5 mm region receiving ≥ 5 Gy) demonstrated the highest single-modality performance (AUC = 0.72). Further integration of clinical, DVH, and DLR_V5Gy_ features via a stacking ensemble (SE) model yielded the best overall results (AUC = 0.76, Recall = 0.70, F1 score = 0.60). These findings highlight the critical role of dose-guided deep features and multimodal fusion in improving clinical toxicity prediction. Importantly, the consistent superiority of ensemble models across all feature combinations underscores their robustness in mitigating overfitting and enhancing generalizability, which is consistent with previous findings by Wu et al. [20], who demonstrated that ensemble strategies outperform single classifiers in RD prediction tasks.

The ablation comparison (Table 3) revealed that while GBDT and RF individually achieved moderate accuracy (AUC ≈ 0.50–0.73), integration through the stacking ensemble consistently improved overall discrimination (AUC = 0.76, F1 = 0.60). These findings demonstrate that each component contributes complementary decision boundaries—GBDT capturing nonlinear dependencies, RF enhancing robustness, and LR improving calibration—thereby validating the ensemble’s synergistic effect.

The design of the input regions had a decisive effect on the model performance. DLR_Original_ and DLR_Skin5mm_ achieved only modest AUCs of 0.74 and 0.72, with Grad-CAM visualizations revealing dispersed attention over irrelevant thoracic structures, which could introduce clinical misclassification risks. DLR_PTV100%_ achieved comparable performance (AUC = 0.74) by focusing on the target volume, although residual deep-tissue signals decreased its specificity. While the AUCs of the DLR-based models were similar overall, DLR_V5Gy_ achieved higher recall and exhibited more clinically consistent attention patterns. Grad-CAM attention for DLR_V5Gy_ was concentrated on the chest wall and subcutaneous skin—regions clinically known to be at high risk for RD. Moreover, these heatmaps strongly overlapped with moderate- to high-dose regions (≥ 35 Gy), reinforcing the consistency between imaging-derived features and DVH analysis. Compared with prior studies that used fixed ROIs or whole-breast inputs [8], our dose–threshold ROI design (V5Gy) effectively guided the model toward clinically relevant structures, improving both accuracy and interpretability. This observation is consistent with that of Jeong et al. [19], who noted the limitations of conventional HCR-based ROI strategies, and Bagherpour et al. [29], who emphasized dose-driven region definitions for toxicity modeling.

Model interpretability further supported these conclusions. SHAP analysis revealed that mid-to-deep convolutional features (e.g., Conv2d_5_66, Conv2d_13_316) were the dominant drivers of prediction, whereas additional features such as Conv2d_8_175, Conv2d_12_51, and Conv2d_9_62 provided stable contributions, indicating that diverse deep-layer representations are crucial for capturing RD-relevant patterns. Clinical features such as age, although less impactful, were consistently retained by Boruta-SHAP, suggesting that they offer complementary signals to imaging features and enhance model stability. This synergy between radiomic features and structured variables echoes findings by Feng et al. [30] and Nie et al. [31] reported that radiomic features dominate toxicity prediction, but nonimaging features can contribute supportive information. Together, the combination of Grad-CAM and SHAP provided transparency, confirmed the biological plausibility of the model, and strengthened clinicians’ trust in AI-assisted tools.

Grad-CAM Clinical Implications: Grad-CAM visualization provided further clinical insight into the spatial attention of different ROI designs. While the DLR_Original_ and DLR_Skin5mm_ models exhibited dispersed activations involving nonirradiated thoracic regions, reducing specificity, and DLR_PTV100%_ focused on tumor boundaries and deeper tissues, their correspondence to RD-prone regions remained limited. In contrast, DLR_V5Gy_ consistently localized attention to the skin and subcutaneous layer from shallow to deeper convolutional layers, aligning closely with medium- to low-dose regions that are clinically recognized as RD high-risk areas. This highlights three key implications: (1) anatomical correspondence, as the skin and subcutaneous tissue represent the most common RD sites [32,33]; (2) dose dependency, with skin reactions showing a dose–dependent relationship with the cumulative radiation dose, supporting the dose–response nature of RD [34,35]; and (3) explainability, as the consistency between AI-derived heatmaps and clinical RD patterns enhances model transparency and physician trust. Compared with Raghavan et al. [36], where conventional Grad-CAM produced broad and inconsistent breast activations, and Liang et al. [37], which primarily demonstrated dose-related correlations without anatomical specificity, the DLR_V5Gy_ approach in the present study uniquely integrates anatomical and dosimetric guidance, providing superior clinical interpretability alongside predictive performance.

Compared with previous studies by Lee et al. (AUC = 0.83) [7] and Xiang et al. (AUC = 0.82) [8], the present study yielded a slightly lower cross-validated AUC (0.72) for the DLR_V5Gy_-only model. This difference likely reflects the use of a more rigorous nested cross-validation (NCV) framework, which provides a conservative and unbiased estimation of generalization performance. Nevertheless, the methodological rationale of the proposed DLR_V5Gy_ approach remains distinct and robust.

Unlike earlier methods that extracted features from fixed or anatomically defined regions—such as the entire breast, superficial skin, or PTV-based ROIs—the DLR_V5Gy_ design introduces a dose-guided ROI, defined as the intersection between the subcutaneous layer within 5 mm of the skin and the isodose volume receiving ≥ 5 Gy. This region better represents the clinically irradiated skin zone, reducing the dilution effect from non-irradiated tissue and improving the biological and dosimetric specificity of extracted features.

Furthermore, Grad-CAM visualizations confirmed that model attention was concentrated in dose-exposed skin areas consistent with radiation-induced toxicity patterns, reinforcing the interpretability and biological plausibility of the framework. When combined with clinical and DVH features in the stacking ensemble configuration, the model’s predictive performance improved further (AUC = 0.76), supporting the value of multimodal fusion in achieving stable, interpretable, and clinically relevant predictions of radiation dermatitis risk.

The inclusion of DCA and clinical utility metrics further underscores the practical relevance of the proposed model. The DCA results indicated consistent net benefit across a wide range of probability thresholds, suggesting that the stacking ensemble could assist clinicians in identifying patients at higher risk with fewer unnecessary interventions. Moreover, the improved PPV, NPV, and balanced false positive/negative rates confirm the model’s potential to support evidence-based decision-making in real-world radiotherapy management.

Several limitations warrant consideration. First, this was a single-center, retrospective study with 148 patients, which may limit its external generalizability. Interestingly, while the homogeneity of imaging protocols and planning systems (Pinnacle and Eclipse) improved training consistency, such uniformity may also limit performance when applied to heterogeneous external datasets. Second, RD grading was based on physician assessment according to the RTOG criteria, which may introduce subjective bias. Third, the high dimensionality of DLR features (4224 variables) poses overfitting risks; although our two-stage feature selection (ANOVA + Boruta-SHAP) effectively reduces redundancy, this step remains challenging. Finally, class imbalance (33% RD incidence) may bias learning toward majority cases, requiring careful handling by resampling strategies.

Despite these limitations, this study has several distinctive strengths. To our knowledge, this is the first work to propose the DLR_V5Gy_ ROI strategy, which directly embeds dose thresholds into deep feature learning, thereby combining anatomical and dosimetric information in a clinically meaningful way. Furthermore, the integration of SHAP and Grad-CAM provided dual interpretability—both feature-level importance and spatial relevance—ensuring that predictions align with clinical knowledge and dose–toxicity relationships.

The clinical implications of this work are considerable. The proposed model can be implemented during treatment planning to identify high-risk patients, enabling physicians to adjust skin dose constraints proactively. For those flagged as high risk, enhanced prophylactic skincare and closer monitoring could be applied to mitigate severe toxicity and reduce treatment interruptions. Embedding the model into radiotherapy planning systems could provide real-time decision support, aligning with the goals of precision oncology to improve the safety, adherence, and cost-effectiveness of breast cancer radiotherapy. Although formal clinician evaluation of the model’s predictions was not performed in this study, the explainable ensemble framework provides SHAP-based interpretability that aligns with clinical reasoning. Future studies will incorporate direct clinician assessment of the model outputs to confirm their clinical relevance and applicability in real-world decision-making.

The proposed ensemble model can be incorporated into existing radiotherapy workflows by integrating its predictive outputs into treatment planning and oncology information systems. Such integration could enable early identification of high-risk patients, assist in dose optimization, and inform personalized follow-up or prophylactic strategies. The explainable SHAP-based outputs further enhance clinical transparency, allowing oncologists to understand the influence of key features on risk prediction. Future implementation will require prospective validation and workflow integration within hospital information systems.

Potential biases inherent to the retrospective cohort were carefully controlled through both data-level and model-level strategies. The synthetic minority oversampling technique combined with the edit nearest neighbors (SMOTE-ENN) was applied only within the training folds to prevent data leakage, ensuring balanced class representation during model training. Moreover, the nested cross-validation framework minimized sampling bias and overfitting, providing a reliable and generalizable estimation of model performance despite the retrospective design.

Future directions include multicenter and cross-platform validation to improve generalizability, prospective trials to evaluate clinical utility, and expansion to other cancer types, such as head and neck malignancies, where RD risk is also substantial. Additionally, the integration of other modalities (MRI, PET, surface imaging) and genomic data could further increase the predictive accuracy. Finally, coupling this framework with dose optimization algorithms could lead to the development of “RD-aware” planning systems, enabling real-time, patient-specific toxicity mitigation strategies. While this study employed ImageNet-pretrained VGG16 for deep feature extraction, future extensions will include domain-specific pretrained models (e.g., RadImageNet, Med3D) and CNNs trained from scratch to further evaluate the impact of different pretraining strategies on radiation dermatitis prediction.

## 5. Conclusions

We developed a hybrid ensemble model that integrates DLR, HCR, clinical features, and DVH parameters to predict RD grades ≥ 2 in breast cancer patients treated with VMAT. Compared with HCR, DLR features provided stronger predictive capability, particularly when the dose-guided DLR_V5Gy_ ROI was applied, which emphasized subcutaneous regions exposed to ≥ 5 Gy and effectively captured clinically relevant toxicity patterns.

The integration of DLR with clinical and DVH variables further enhanced the predictive reliability, with the stacking ensemble achieving the best overall discrimination across the AUC, accuracy, and recall. Explainability techniques (SHAP and Grad-CAM) confirmed that the predictions were grounded in dose-related and anatomical features, reinforcing the biological plausibility and clinical trustworthiness of the model.

Overall, this framework demonstrated high accuracy, robustness, and interpretability, offering practical potential for the early identification of high-risk patients, individualized prevention strategies, and treatment planning optimization. Future multicenter and prospective validation will be critical to enable clinical translation, ultimately paving the way toward explainable AI–driven decision-support systems in radiotherapy.

## Figures and Tables

**Table 1:** Clinical characteristics of breast cancer patients.

Patient Characteristics	Total	Without RD	With RD	*p*
	*N* = 148 (100%)	*n* = 99 (66.9%)	*n* = 49 (33.1%)	
*Age*				0.035
Mean	56	55	59	
*BMI*				0.346
Mean	24.73	24.46	25.29	
*Laterality*				0.323
Left	70 (47.3)	44 (29.7)	26 (17.6)	
Right	78 (52.7)	55 (37.2)	23 (15.5)	
*Surgery Type*				0.861
TM/MRM	29 (19.6)	19 (12.8)	10 (6.8)	
BCS	119 (80.4)	80 (54.1)	39 (26.4)	
*AJCC Stage*				0.776
0	34 (23.0)	24 (16.2)	10 (6.8)	
1	46 (31.1)	31 (20.9)	15 (10.1)	
2	48 (32.4)	32 (21.6)	16 (10.8)	
3	17 (11.5)	11 (7.4)	6 (4.1)	
4	3 (2.0)	1 (0.7)	2 (1.4)	
*SCF*				0.077
No	110 (74.3)	78 (52.7)	32 (21.6)	
Yes	38 (25.7)	21 (14.2)	17 (11.5)	
*IMN*				0.033
No	115 (77.7)	82 (55.4)	33 (22.3)	
Yes	33 (22.3)	17 (11.5)	16 (10.8)	
*Chemotherapy*				0.600
No	74 (50.0)	51 (34.5)	23 (15.5)	
Yes	74 (50.0)	48 (32.4)	26 (17.6)	

Abbreviation: RD, Radiation Dermatitis; BMI, Body Mass Index; TM, Total Mastectomy; MRM, Modified Radical Mastectomy; BCS, Breast-Conserving Surgery; AJCC, American Joint Committee on Cancer; SCF, Supraclavicular Fossa; IMN, Internal Mammary Nodes.

**Table 2:** Dose-volume histogram (DVH) features of breast cancer patients.

DVH Feature	Total *N* = 148 (100%)	Without RD	With RD	*p*
		*n* = 99 (67%)	*n* = 49 (33%)	
	Mean ± SD (cc)	Mean ± SD (cc)	Mean ± SD (cc)	
Skin5mm V_5Gy_	325.0 ± 120.9	312.7 ± 123.6	349.9 ± 112.3	0.078
Skin5mm V_10Gy_	258.1 ± 89.3	248.6 ± 90.9	277.2 ± 83.7	0.067
Skin5mm V_15Gy_	225.9 ± 74.0	218.0 ± 76.3	241.8 ± 67.0	0.064
Skin5mm V_20Gy_	204.9 ± 62.2	198.2 ± 63.8	218.3 ± 57.1	0.065
**Skin5mm V_25Gy_**	**187.4 ± 53.8**	**181.26 ± 55.1**	**199.8 ± 49.4**	**0.048**
**Skin5mm V_30Gy_**	**171.8 ± 46.8**	**166.5 ± 47.8**	**182.5 ± 43.4**	**0.049**
Skin5mm V_35Gy_	156.9 ± 41.0	152.3 ± 41.8	166.1 ± 38.2	0.054
Skin5mm V_40Gy_	141.4 ± 36.6	137.5 ± 37.3	149.3 ± 37.1	0.065
**Skin5mm V_45Gy_**	**118.2 ± 31.1**	**114.6 ± 31.1**	**125.4 ± 30.1**	**0.046**
Skin5mm V_50Gy_	47.6 ± 24.6	46.3 ± 24.8	50.2 ± 24.2	0.366
**PTV_100%_**	**703.1 ± 387.6**	**648.0 ± 374.3**	**814.5 ± 394.1**	**0.013**
PTV_105%_	69.2 ± 133.7	57.2 ± 80.0	93.5 ± 202.1	0.121

Values are expressed as the mean ± SD. Bold + gray highlight indicates *p* < 0.05. Abbreviation: V_XGy_, Volume in cubic centimeters of skin receiving x dose of Gray; PTV_X%_, Planning Target Volume Receiving x% of the Prescription Dose; RD, Radiation Dermatitis; cc, cubic centimeter.

**Table 3:** Summary of classification metrics (mean ± 95% CI), including AUC, accuracy, recall, specificity, precision (PPV), NPV, and F1-score, for AI models across multiple feature combinations.

Group	Feature	Model	AUC	ACC	Recall	Specificity	NPV	PPV	F1-Score
1	Clinical DVH	LR	**0.62 ± 0.13**	0.33 ± 0.02	**1.00 ± 0.00**	0.00 ± 0.00	0.00 ± 0.00	0.33 ± 0.02	**0.50 ± 0.02**
		RF	0.58 ± 0.17	0.54 ± 0.17	0.70 ± 0.38	0.47 ± 0.35	**0.62 ± 0.45**	**0.39 ± 0.13**	0.48 ± 0.19
		GBDT	0.50 ± 0.00	0.33 ± 0.02	**1.00 ± 0.00**	0.00 ± 0.00	0.00 ± 0.00	0.33 ± 0.02	**0.50 ± 0.02**
		Ensemble	0.61 ± 0.19	0.46 ± 0.14	0.71 ± 0.42	0.33 ± 0.43	0.43 ± 0.49	0.35 ± 0.03	0.44 ± 0.14
2	HCR	LR	**0.68 ± 0.20**	0.33 ± 0.02	**1.00 ± 0.00**	0.00 ± 0.00	0.00 ± 0.00	0.33 ± 0.02	0.50 ± 0.02
		RF	0.56 ± 0.10	0.51 ± 0.23	0.73 ± 0.31	0.39 ± 0.48	0.44 ± 0.51	0.43 ± 0.20	0.50 ± 0.10
		GBDT	0.54 ± 0.06	0.33 ± 0.02	0.96 ± 0.11	0.02 ± 0.06	0.10 ± 0.28	0.33 ± 0.02	0.49 ± 0.03
		Ensemble	0.66 ± 0.11	0.66 ± 0.14	0.47 ± 0.24	0.76 ± 0.12	0.75 ± 0.11	0.49 ± 0.21	0.48 ± 0.22
3	DLR_Original_	LR	0.68 ± 0.06	0.65 ± 0.15	**0.63 ± 0.14**	0.66 ± 0.27	**0.78 ± 0.05**	0.51 ± 0.15	0.55 ± 0.09
		RF	0.72 ± 0.09	**0.70 ± 0.11**	0.57 ± 0.10	0.77 ± 0.17	**0.78 ± 0.06**	**0.58 ± 0.15**	**0.57 ± 0.10**
		GBDT	0.70 ± 0.11	0.66 ± 0.14	0.51 ± 0.07	0.73 ± 0.20	0.74 ± 0.07	0.52 ± 0.18	0.50 ± 0.11
		Ensemble	**0.74 ± 0.11**	0.68 ± 0.11	0.47 ± 0.06	**0.78 ± 0.16**	0.74 ± 0.05	0.55 ± 0.21	0.50 ± 0.09
4	DLR_Skin5mm_	LR	0.71 ± 0.10	0.66 ± 0.14	**0.62 ± 0.22**	0.69 ± 0.23	0.79 ± 0.09	0.52 ± 0.19	0.55 ± 0.15
		RF	0.72 ± 0.13	0.69 ± 0.15	0.53 ± 0.18	0.77 ± 0.17	0.77 ± 0.10	0.55 ± 0.23	0.54 ± 0.19
		GBDT	0.69 ± 0.18	0.69 ± 0.14	0.55 ± 0.25	0.76 ± 0.17	0.78 ± 0.11	0.55 ± 0.27	0.54 ± 0.21
		Ensemble	**0.72 ± 0.16**	**0.74 ± 0.11**	0.53 ± 0.18	**0.84 ± 0.13**	**0.79 ± 0.08**	**0.64 ± 0.25**	**0.57 ± 0.18**
5	DLR_PTV100%_	LR	0.68 ± 0.20	0.66 ± 0.20	0.64 ± 0.13	0.67 ± 0.26	**0.77 ± 0.13**	**0.52 ± 0.23**	**0.57 ± 0.19**
		RF	0.69 ± 0.15	0.54 ± 0.12	**0.72 ± 0.38**	0.46 ± 0.20	0.82 ± 0.21	0.38 ± 0.12	0.49 ± 0.19
		GBDT	0.69 ± 0.08	0.63 ± 0.08	0.45 ± 0.19	0.72 ± 0.06	0.73 ± 0.08	0.43 ± 0.11	0.44 ± 0.15
		Ensemble	**0.70 ± 0.05**	**0.67 ± 0.06**	0.49 ± 0.11	**0.76 ± 0.09**	0.75 ± 0.05	0.51 ± 0.10	0.49 ± 0.08
6	DLR_V5Gy_	LR	0.69 ± 0.05	0.62 ± 0.09	0.78 ± 0.16	0.53 ± 0.12	**0.83 ± 0.13**	0.46 ± 0.06	0.57 ± 0.08
		RF	0.72 ± 0.06	0.61 ± 0.07	0.78 ± 0.16	0.53 ± 0.09	**0.83 ± 0.10**	0.45 ± 0.06	0.57 ± 0.08
		GBDT	0.72 ± 0.10	0.33 ± 0.02	**1.00 ± 0.00**	0.00 ± 0.00	0.00 ± 0.00	0.33 ± 0.02	0.50 ± 0.02
		Ensemble	**0.72 ± 0.05**	**0.65 ± 0.07**	0.70 ± 0.12	**0.63 ± 0.14**	0.81 ± 0.05	**0.49 ± 0.06**	**0.57 ± 0.05**
7	Clinical DVH HCR	LR	0.64 ± 0.09	0.59 ± 0.17	0.65 ± 0.15	**0.56 ± 0.26**	0.74 ± 0.14	**0.44 ± 0.12**	**0.52 ± 0.12**
		RF	0.67 ± 0.16	0.55 ± 0.22	**0.69 ± 0.24**	0.4 ± 0.42	0.59 ± 0.42	**0.44 ± 0.16**	0.51 ± 0.09
		GBDT	0.56 ± 0.10	0.41 ± 0.11	0.86 ± 0.21	0.18 ± 0.26	0.43 ± 0.50	0.34 ± 0.03	0.49 ± 0.03
		Ensemble	**0.68 ± 0.15**	**0.55 ± 0.11**	0.67 ± 0.35	0.48 ± 0.26	**0.80 ± 0.19**	0.39 ± 0.09	0.48 ± 0.16
8	Clinical DVH DLR_Original_	LR	0.69 ± 0.04	0.64 ± 0.13	**0.61 ± 0.18**	0.65 ± 0.26	0.77 ± 0.04	0.49 ± 0.12	**0.53 ± 0.08**
		RF	0.69 ± 0.03	0.68 ± 0.13	0.53 ± 0.20	0.75 ± 0.26	0.76 ± 0.04	**0.56 ± 0.14**	0.52 ± 0.10
		GBDT	0.69 ± 0.03	0.59 ± 0.15	0.67 ± 0.25	0.55 ± 0.24	**0.78 ± 0.18**	0.44 ± 0.11	0.52 ± 0.12
		Ensemble	**0.70 ± 0.04**	**0.67 ± 0.06**	0.45 ± 0.16	**0.78 ± 0.16**	0.74 ± 0.02	0.53 ± 0.09	0.47 ± 0.06
9	Clinical DVH DLR_Skin5mm_	LR	0.69 ± 0.08	0.62 ± 0.09	0.69 ± 0.09	0.58 ± 0.19	0.79 ± 0.04	0.46 ± 0.09	0.55 ± 0.07
		RF	0.70 ± 0.10	0.55 ± 0.09	0.78 ± 0.20	0.43 ± 0.19	**0.81 ± 0.09**	0.41 ± 0.05	0.53 ± 0.08
		GBDT	**0.73 ± 0.07**	0.48 ± 0.11	**0.92 ± 0.16**	0.26 ± 0.23	0.73 ± 0.52	0.39 ± 0.03	**0.54 ± 0.03**
		Ensemble	0.66 ± 0.12	**0.66 ± 0.11**	0.49 ± 0.25	**0.74 ± 0.11**	0.75 ± 0.10	**0.47 ± 0.16**	0.48 ± 0.20
10	Clinical DVH DLR_PTV100_	LR	0.66 ± 0.18	0.58 ± 0.11	0.75 ± 0.25	0.50 ± 0.21	**0.84 ± 0.15**	0.43 ± 0.08	0.54 ± 0.11
		RF	0.66 ± 0.18	0.56 ± 0.12	0.78 ± 0.13	0.45 ± 0.14	0.79 ± 0.14	0.41 ± 0.09	0.54 ± 0.10
		GBDT	0.66 ± 0.09	0.55 ± 0.09	**0.78 ± 0.27**	0.44 ± 0.17	0.84 ± 0.19	0.40 ± 0.07	0.53 ± 0.11
		Ensemble	**0.67 ± 0.10**	**0.58 ± 0.13**	0.76 ± 0.22	**0.50 ± 0.25**	0.83 ± 0.14	**0.44 ± 0.10**	**0.55 ± 0.10**
11	Clinical DVH DLR_V5Gy_	LR	0.69 ± 0.08	0.62 ± 0.09	0.69 ± 0.09	0.58 ± 0.16	0.79 ± 0.04	0.46 ± 0.09	0.55 ± 0.07
		RF	0.73 ± 0.04	0.61 ± 0.07	0.74 ± 0.14	0.55 ± 0.12	0.81 ± 0.08	0.45 ± 0.07	0.56 ± 0.06
		GBDT	0.73 ± 0.09	0.51 ± 0.05	0.88 ± 0.16	0.32 ± 0.15	**0.88 ± 0.14**	0.39 ± 0.02	0.54 ± 0.02
		**Ensemble**	**0.76 ± 0.06**	**0.70 ± 0.09**	**0.68 ± 0.16**	**0.71 ± 0.15**	**0.82 ± 0.08**	**0.55 ± 0.10**	**0.60 ± 0.08**

Best performance per feature combination is highlighted in bold for ease of comparison. Abbreviation: SHAP, SHapley Additive exPlanations; RD, Radiation Dermatitis; DVH, Dose-Volume Histogram; HCR, Handcrafted Radiomics; DLR, Deep Learning Radiomics; LR, Logistic Regression; RF, Random Forest; GBDT, Gradient Boosting Decision Tree; AUC, Area Under the ROC Curve; ROC, Receiver Operating Characteristic; ACC, Accuracy; NPV, Negative Predictive Value; PPV, Positive Predictive Value; 95% CI, 95% Confidence Interval.

## Data Availability

The datasets generated and/or analyzed during the current study are not publicly available owing to institutional ethical restrictions and patient privacy regulations but are available from the corresponding authors (Pei-Ju Chao, pjchao99@gmail.com; Yu-Wei Lin, marklin1108@gmail.com; Tsair-Fwu Lee, tflee@nkust.edu.tw) upon reasonable request and with appropriate ethical approval from the Kaohsiung Veterans General Hospital Institutional Review Board (IRB No. KSVGH23-CT12-09, Approval Date: 5 December 2023). Supporting data, including feature extraction scripts and model performance metrics, are provided in the Supplementary Materials. The feature extraction scripts and performance data are provided in the Supplementary Materials.

## Supplementary Materials

The following supporting information can be downloaded at: https://www.mdpi.com/article/10.3390/cancers17233767/s1, Table S1. HCR features and feature categories. Table S2. Definitions and formulas for model performance metrics. Table S3. Overview of 11 feature combinations and representative features. Table S4. Brier scores across feature combinations and models. Table S5. Friedman test results indicating statistically significant differences among models. Figure S1. Hyperparameter sensitivity analysis of the stacking ensemble model using the feature combination Clinical + DVH + DLR_V5Gy_. Figure S2. Calibration curves of the ensemble model across three representative feature sets. Figure S3. Decision curve analyses (DCA) of the ensemble model across three representative feature sets.
