# Supplementary material for: Dose-Guided Hybrid AI Model with Deep and Handcrafted Radiomics for Explainable Radiation Dermatitis Prediction in Breast Cancer VMAT
Source: Cancers (Basel). 2025 Nov 26;17(23):3767. doi: 10.3390/cancers17233767 (PMC12691004; doi:10.3390/cancers17233767)
Supplement: Supplementary file 1 [file cancers-17-03767-s001.zip › cancers-3902657-supplementary.pdf]

Supplementary Tables

Supplementary Table S1. HCR features and feature categories

Shape Features	First-order Features
	1. Energy
	2. Total Energy
1. Mesh Volume	3. Entropy
2. Voxel Volume	4. Minimum
3. Surface Area	5. 10Percentile
4. Surface Area to Volume Ratio	6. 90Percentile
5. Sphericity	7. Maximum
6. Maximum 3D Diameter	8. Mean
7. Maximum 2D Diameter Slice	9. Median
8. Maximum 2D Diameter Column	10. Interquartile Range
9. Maximum 2D Diameter Row	11. Range
10. Major Axis Length	12. Mean Absolute Deviation
11. Minor Axis Length	13. Robust Mean Absolute Deviation
12. Least Axis Length	14. Root Mean Squared
13. Elongation	15. Skewness
14. Flatness	16. Kurtosis
	17. Variance
	18. Uniformity
**Texture Features**	
GLCM	GLSZM

1. Autocorrelation	
2. Joint Average	
3. Cluster Prominence	
4. Cluster Shade	1. Small Area Emphasis
5. Cluster Tendency	2. Large Area Emphasis
6. Contrast	3. Gray Level Non-Uniformity
7. Correlation	4. Gray Level Non-Uniformity Normalized
8. Difference Average	5. Size Zone Non-Uniformity
9. Difference Entropy	6. Size Zone Non-Uniformity Normalized
10. Difference Variance	7. Zone Percentage
11. Joint Energy	8. Gray Level Variance
12. Joint Entropy	9. Zone Variance
13. Informational Measure of Correlation 1	10. Zone Entropy
14. Informational Measure of Correlation 2	11. Low Gray Level Zone Emphasis
15. Inverse Difference Moment	12. High Gray Level Zone Emphasis
16. Inverse Difference Moment Normalized	13. Small Area Low Gray Level Emphasis
17. Inverse Difference	14. Large Area High Gray Level Emphasis
18. Inverse Difference Normalized	15. Large Area Low Gray Level Emphasis
19. Inverse Variance	16. Small Area High Gray Level Emphasis
20. Maximum Probability	
21. Sum Entropy	
22. Sum Squares	
GLRLM	GLDM
1. Short Run Emphasis	1. Small Dependence Emphasis
2. Long Run Emphasis	2. Large Dependence Emphasis
3. Gray Level Non-Uniformity	3. Gray Level Non-Uniformity
4. Gray Level Non-Uniformity Normalized	4. Dependence Non-Uniformity
5. Run Length Non-Uniformity	5. Dependence Non-Uniformity Normalized
6. Run Length Non-Uniformity Normalized	6. Gray Level Variance
7. Run Percentage	7. Dependence Variance
8. Gray Level Variance	8. Dependence Entropy

9. Run Variance	9. Low Gray Level Emphasis
10. Run Entropy	10. High Gray Level Emphasis
11. Low Gray Level Run Emphasis	11. Small Dependence Low Gray Level Emphasis
12. High Gray Level Run Emphasis	12. Small Dependence High Gray Level Emphasis
13. Short Run Low Gray Level Emphasis	13. Large Dependence Low Gray Level Emphasis
14. Short Run High Gray Level Emphasis	14. Large Dependence High Gray Level Emphasis
15. Long Run Low Gray Level Emphasis	
16. Long Run High Gray Level Emphasis	
NGTDM	
1. Coarseness	
2. Contrast	
3. Busyness	
4. Complexity	
5. Strength	

Shape Features: Mesh Volume, Voxel Volume, Surface Area, Surface Area to Volume Ratio, Sphericity, Maximum 3D Diameter, Maximum 2D Diameter Slice, Maximum 2D Diameter Column, Maximum 2D Diameter Row, Major Axis Length, Minor Axis Length, Least Axis Length, Elongation, Flatness.

First-order Features: Energy, Total Energy, Entropy, Minimum, 10th Percentile, 90th Percentile, Maximum, Mean, Median, Interquartile Range, Range, Mean Absolute Deviation, Robust Mean Absolute Deviation, Root Mean Squared, Skewness, Kurtosis, Variance, Uniformity.

Texture Features:

GLCM (Gray Level Co-occurrence Matrix): Autocorrelation, Joint Average, Cluster Prominence, Cluster Shade, Cluster Tendency, Contrast, Correlation, Difference Average, Difference Entropy, Difference Variance, Joint Energy, Joint Entropy, Informational Measure of Correlation 1, Informational Measure of Correlation 2, Inverse Difference Moment, Inverse Difference Moment Normalized, Inverse Difference, Inverse Difference Normalized, Inverse Variance, Maximum Probability, Sum Entropy, Sum of Squares.

GLSZM (Gray Level Size Zone Matrix): Small Area Emphasis, Large Area Emphasis, Gray Level Non-Uniformity, Gray Level Non-Uniformity Normalized, Size Zone Non-Uniformity, Size Zone Non-Uniformity Normalized, Zone Percentage, Gray Level Variance, Zone Variance, Zone Entropy, Low Gray Level Zone Emphasis, High Gray Level Zone Emphasis, Small Area Low Gray

Level Emphasis, Large Area High Gray Level Emphasis, Large Area Low Gray Level Emphasis, Small Area High Gray Level Emphasis.

GLRLM (Gray Level Run Length Matrix): Short Run Emphasis, Long Run Emphasis, Gray Level Non-Uniformity, Gray Level Non-Uniformity Normalized, Run Length Non-Uniformity, Run Length Non-Uniformity Normalized, Run Percentage, Gray Level Variance, Run Variance, Run Entropy, Low Gray Level Run Emphasis, High Gray Level Run Emphasis, Short Run Low Gray Level Emphasis, Short Run High Gray Level Emphasis, Long Run Low Gray Level Emphasis, Long Run High Gray Level Emphasis.

GLDM (Gray Level Dependence Matrix): Small Dependence Emphasis, Large Dependence Emphasis, Gray Level Non-Uniformity, Dependence Non-Uniformity, Dependence Non-Uniformity Normalized, Gray Level Variance, Dependence Variance, Dependence Entropy, Low Gray Level Emphasis, High Gray Level Emphasis, Small Dependence Low Gray Level Emphasis, Small Dependence High Gray Level Emphasis, Large Dependence Low Gray Level Emphasis, Large Dependence High Gray Level Emphasis.

NGTDM (Neighborhood Gray Tone Difference Matrix): Coarseness, Contrast, Busyness, Complexity, Strength.

Note: GLCM, Gray Level Co-occurrence Matrix; GLSZM, Gray Level Size Zone Matrix; GLRLM, Gray Level Run Length Matrix; GLDM, Gray Level Dependence Matrix; NGTDM, Neighborhood Gray Tone Difference Matrix.

Supplementary Table S2. Definitions and formulas for model performance metrics

**Metric**	**Formula**	**Definition**
AUC (Area Under the ROC Curve)	$\sum (FPR_{i+1} - FPR_i) \frac{(TPR_{i+1} + TPR_i)}{2}$	Area under the ROC curve, reflecting the model's discriminative ability.
Accuracy	$\frac{TP + \mathrm{TN}}{TP + \mathrm{TN} + \mathrm{FP} + FN}$	Overall proportion of correctly classified cases.
Recall (Sensitivity)	$\frac{TP}{TP + FN}$	True positive rate.
Specificity	$\frac{T\mathrm{N}}{T\mathrm{N} + F\mathrm{P}}$	True negative rate.
PPV (Positive Predictive Value)	$\frac{TP}{TP + F\mathrm{P}}$	Probability that a predicted positive case is correct.

NPV (Negative Predictive Value)	$\frac{TN}{TN + FN}$	Probability that a predicted negative case is correct.
F1-score	$2\frac{(\text{Precision} \times Recall)}{(\text{Precision} + Recall)} = \frac{2TP}{2TP + \text{FP} + FN}$	Harmonic mean of Precision and Recall.

Note: TP, True Positive; TN, True Negative; FP, False Positive; FN, False Negative; TPR, True Positive Rate; FPR, False Positive Rate.

Supplementary Table S3. Overview of 11 feature combinations and representative features

Group	**Feature Set**	Feature Count	Feature
**1**	Clinical<br>DVH	3	Age<br>PTV_100%<br>2500-true5mm
**2**	HCR	8	R_V50_true5mm_original_glszm_LowGrayLevelZoneEmphasis<br>R_V10_true5mm_original_ngtdm_Contrast<br>R_V25_true5mm_original_glszm_GrayLevelNonUniformity<br>R_PTV_100_original_shape_MinorAxisLength<br>R_V50_true5mm_original_ngtdm_Busyness<br>R_V35_true5mm_original_shape_Flatness<br>R_V30_true5mm_original_shape_Flatness<br>R_V10_true5mm_original_firstorder_Minimum
**3**	DLROriginal	6	Conv2d_11_394<br>Conv2d_12_304<br>Conv2d_9_190<br>Conv2d_13_466<br>Conv2d_12_265<br>Conv2d_13_317
**4**	DLRSkin5mm	5	Conv2d_13_391<br>Conv2d_13_298<br>Conv2d_13_13<br>Conv2d_13_154<br>Conv2d_12_272
**5**	DLRPTV100%	6	Conv2d_9_159<br>Conv2d_12_148<br>Conv2d_13_412<br>Conv2d_12_398<br>Conv2d_5_216<br>Conv2d_13_260
**6**	DLRV5Gy	5	Conv2d_8_175<br>Conv2d_12_51<br>Conv2d_13_316<br>Conv2d_5_66<br>Conv2d_9_62

**7**	Clinical<br>DVH<br>HCR	10	R_V50_true5mm_original_glszm_LowGrayLevelZoneEmphasis<br>Age<br>R_V10_true5mm_original_ngtdm_Contrast<br>R_V25_true5mm_original_glszm_GrayLevelNonUniformity<br>R_PTV_100_original_shape_MinorAxisLength<br>R_V50_true5mm_original_ngtdm_Busyness<br>R_V35_true5mm_original_shape_Flatness<br>R_V30_true5mm_original_shape_Flatness<br>R_V10_true5mm_original_firstorder_Minimum<br>R_V45_true5mm_original_glszm_LargeAreaLowGrayLevelEmphasis
8	Clinical<br>DVH<br>DLR_O	11	Conv2d_12_287<br>Conv2d_11_394<br>Conv2d_13_58<br>Conv2d_12_304<br>Conv2d_9_190<br>Conv2d_12_294<br>Conv2d_13_466<br>Conv2d_12_265<br>Conv2d_13_177<br>Conv2d_13_317<br>2500-true5mm
**9**	Clinical<br>DVH<br>DLR_5mm	7	Conv2d_13_50<br>Conv2d_13_391<br>Conv2d_13_298<br>Conv2d_13_13<br>Conv2d_13_154<br>2500-true5mm<br>Conv2d_12_272
**10**	Clinical<br>DVH<br>DLR_PTV100	13	Age<br>Conv2d_9_334<br>Conv2d_9_159<br>Conv2d_6_18<br>Conv2d_12_148<br>Conv2d_11_101<br>Conv2d_5_229<br>Conv2d_13_16<br>Conv2d_12_398<br>Conv2d_5_216<br>Conv2d_13_509<br>Conv2d_13_412<br>Conv2d_13_260
**11**	Clinical<br>DVH<br>DLR_V5Gy	6	Conv2d_8_175<br>Age<br>Conv2d_12_51<br>Conv2d_13_316<br>Conv2d_5_66<br>Conv2d_9_62

Note: DLR, Deep Learning Radiomics; R, Radiomics; DVH, Dose–Volume Histogram; PTV, Planning Target Volume; Skin5mm, 5-mm subcutaneous skin region; V5Gy, subcutaneous region receiving ≥5 Gy dose; O, Original CT; glszm, Gray Level Size Zone Matrix; Neighborhood Gray Tone Difference Matrix; PTV, Planning Target Volume; Conv2d, 2D Convolutional Layer

Supplementary Table S4. Brier scores across feature combinations and models

Group	Feature Set	Model			
		LR	RF	GBDT	Ensemble
1	Clinical<br>DVH	0.270	0.307	0.258	0.298
2	HCR	0.340	0.304	0.330	0.295
3	DLROriginal	0.305	0.237	0.288	0.240
4	DLRSkin5mm	0.267	0.264	0.322	0.320
5	DLRPTV100%	0.273	0.282	0.355	0.272
6	DLRV5Gy	0.319	0.267	0.288	0.249
7	Clinical<br>DVH<br>HCR	0.288	0.258	0.288	0.331
8	Clinical<br>DVH<br>DLR_O	0.293	0.269	0.354	0.312
9	Clinical<br>DVH<br>DLR_5mm	0.249	0.293	0.335	0.340
10	Clinical<br>DVH<br>DLR_PTV100	0.327	0.304	0.322	0.323
11	Clinical<br>DVH<br>DLR_V5Gy	0.280	0.249	0.261	0.249

Note: LR, Logistic Regression; RF, Random Forest; GBDT, Gradient Boosting Decision Tree; DVH, Dose–Volume Histogram; HCR, Handcrafted Radiomics; DLR, Deep Learning Radiomics; O, Original CT

Supplementary Table S5. Friedman test results indicating statistically significant differences among models

Group	Feature Set	Friedman statistic	p-value
**1**	Clinical<br>DVH	1.44	**0.70**
**2**	HCR	0.83	**0.84**
**3**	DLROriginal	6.80	**0.08**
**4**	DLRSkin5mm	3.00	**0.39**
**5**	DLRPTV100%	0.60	**0.90**
**6**	DLRV5Gy	3.24	**0.36**
**7**	Clinical<br>DVH<br>HCR	2.17	**0.54**
**8**	Clinical<br>DVH<br>DLR_O	3.96	**0.27**
**9**	Clinical<br>DVH<br>DLR_5mm	6.12	**0.11**
**10**	Clinical<br>DVH<br>DLR_PTV100	1.08	**0.78**
**11**	Clinical<br>DVH<br>DLR_V5Gy	9.61	**0.02**

Note: DVH, Dose–Volume Histogram; HCR, Handcrafted Radiomics; DLR, Deep Learning Radiomics; O, Original CT

Supplementary Figures

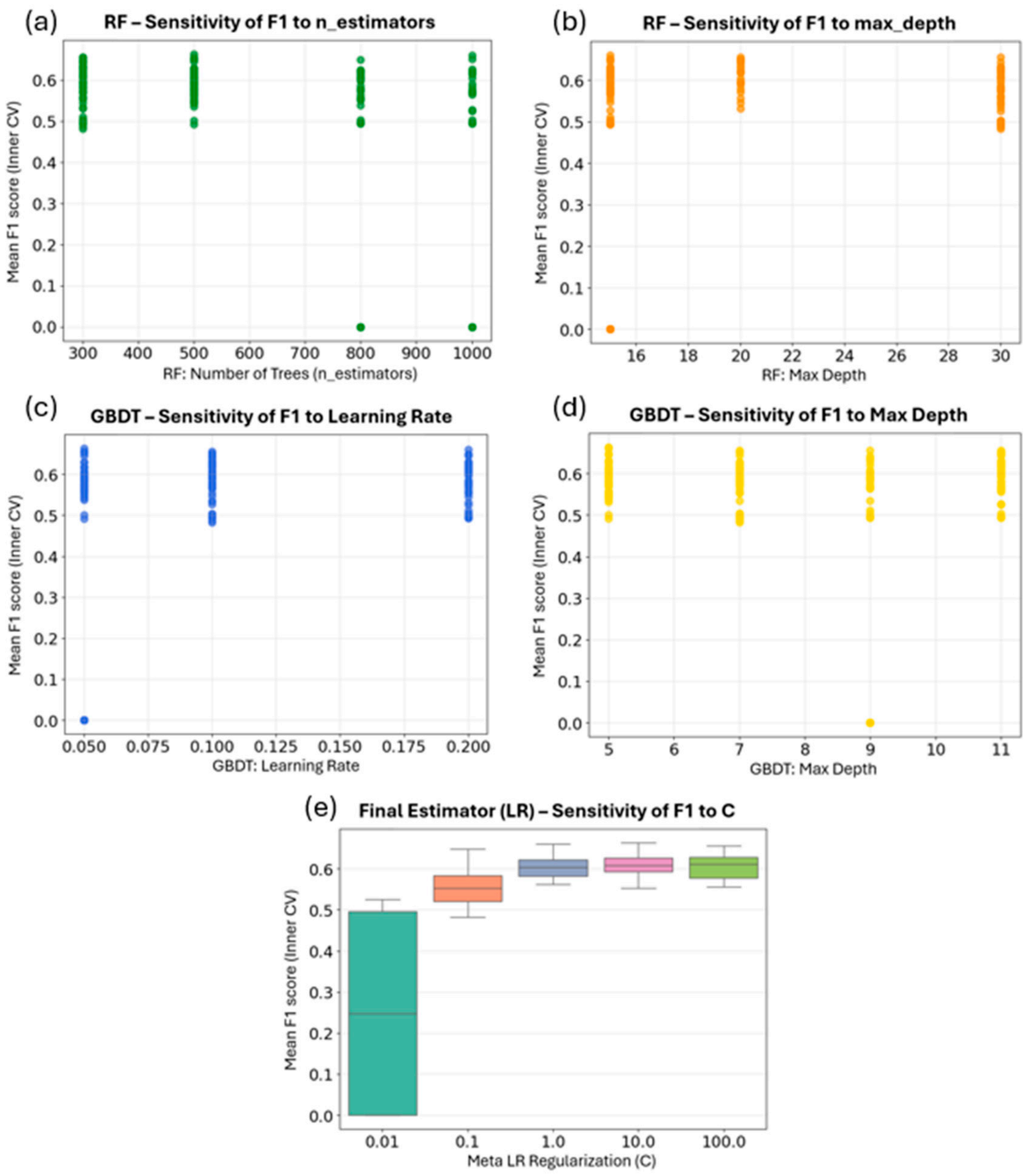

Supplementary Figure S1. Hyperparameter sensitivity analysis of the stacking ensemble model using the feature combination Clinical + DVH + DLRV5Gy.

(a–b) Random Forest (RF): sensitivity of F1 to the number of trees (n_estimators) and maximum tree depth (max_depth).
(c–d) Gradient Boosting Decision Tree (GBDT): sensitivity of F1 to learning rate and maximum depth.
(e) Meta-classifier (Logistic Regression): boxplot showing the relationship between regularization strength (C) and model performance.

The results demonstrate that the ensemble model maintains stable F1 performance across a broad hyperparameter space, supporting its robustness and generalizability.

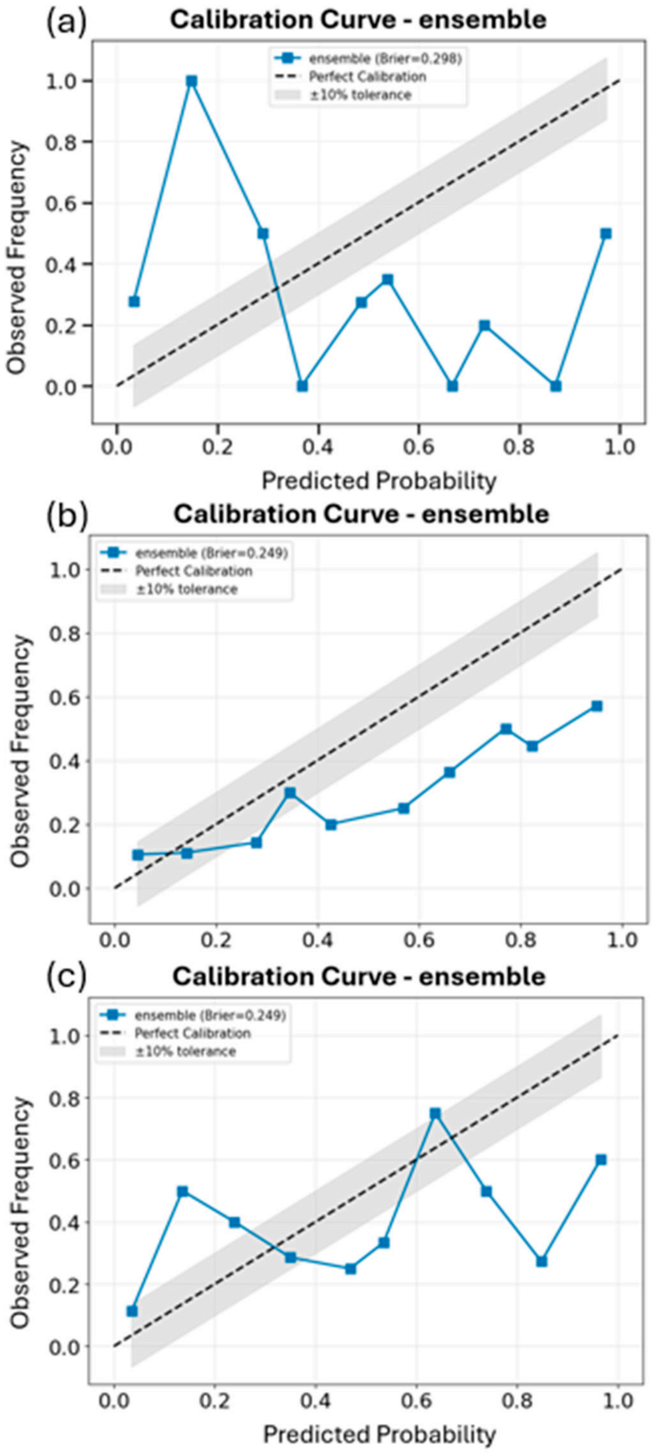

Supplementary Figure S2. Calibration curves of the ensemble model across three representative feature sets.

(a) Baseline combination — Clinical + DVH (Combination 1);

(b) Single radiomics modality — DLRV5Gy (Combination 6);

(c) Multimodal combination — Clinical + DVH + DLRV5Gy (Combination 11).

The dashed diagonal line represents perfect calibration, and the shaded region indicates the ±10% tolerance interval. Lower Brier scores reflect better agreement between predicted probabilities and observed outcomes.

Note: DVH, Dose–Volume Histogram; DLR, Deep Learning Radiomics; V5Gy, subcutaneous region receiving ≥5 Gy dose

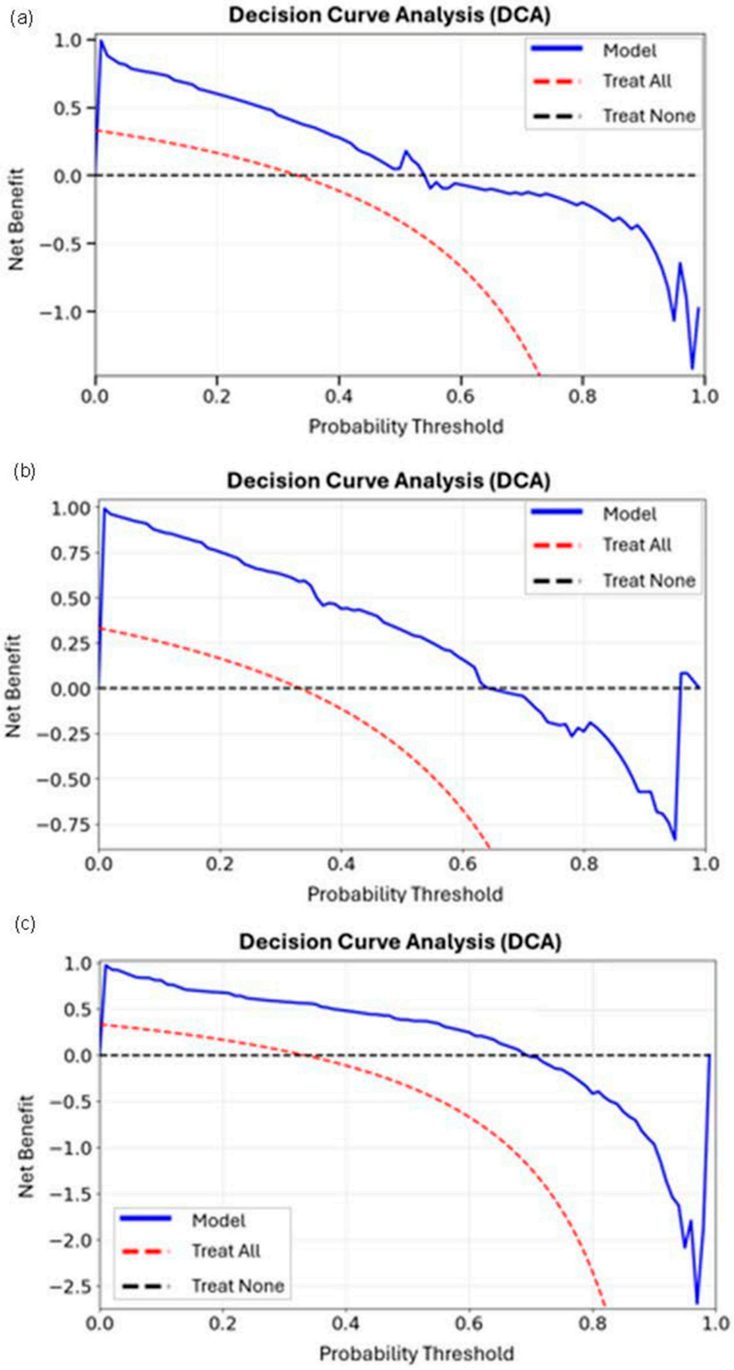

Supplementary Figure S3. Decision curve analyses (DCA) of the ensemble model

across three representative feature sets.

(a) Baseline combination — Clinical + DVH (Combination 1);

(b) Single radiomics modality — DLRV5Gy (Combination 6);

(c) Multimodal combination — Clinical + DVH + DLRV5Gy (Combination 11).

Decision curve analyses illustrate the clinical net benefit of the ensemble model across varying probability thresholds, compared with “treat-all” and “treat-none” strategies. The ensemble model demonstrates consistently higher net benefit across a broad range of thresholds, indicating improved clinical utility.

Note: DVH, Dose–Volume Histogram; DLR, Deep Learning Radiomics; V5Gy, subcutaneous region receiving ≥5 Gy dose

All analyses were conducted using Python 3.8 with scikit-learn and imbalanced-learn. Representative scripts are available via OneDrive:

https://1drv.ms/f/c/3914279715fceea1/EhENhvhhtLlHrObjQtB1nWcB55p9DB48w1fdd_4Mdtkmgg
